# Green chemistry

**DOI:** 10.3762/bjoc.12.273

**Published:** 2016-12-15

**Authors:** Luigi Vaccaro

**Affiliations:** 1 Laboratory of Green Synthetic Organic Chemistry, Dipartimento di Chimica, Biologia e Biotecnologie, Università di Perugia, Via Elce di Sotto, 8 06123 Perugia, Italia

**Keywords:** green chemistry, sustainable chemistry

Since their initial appearance in the scientific literature, the terms "green" and "sustainable" have been increasingly used and are nowadays ubiquitously present in the terminology of several research areas. The seminal origin of what is considered “green chemistry” today might be ascribed to the launch of the Responsible Care^®^ initiative by the American Chemistry Council (ACC) [1] and to the Brundtland report [2]. The concept was then further refined and completed with the Pollution Prevention Act (approved by the American Congress [3]) and the definition of the Anastas and Warner’s 12 principles of green chemistry [4–5]. Very generally, green chemistry may be considered as the scientific and economical context in which academia, industry and government are attempting to converge their efforts for the development of a sustainable civilization.

The first goal of green chemistry is to provide a solid solution to the need for an ex novo design of the existing and necessary chemical processes by primarily considering safety, pollution prevention, waste minimization and energy optimization. To achieve such goals, the necessity of chemists from different areas is evident. Also important is how this novel approach to scientific research has led to a different and hopefully more effective paradigm in the collaboration between industry and academia.

It is obvious that the chemical yield represents just one of the many features that a process must possess to be considered efficient. It is of extreme importance nowadays to consider not only the safety of a chemical procedure, but also the proper selection of solvents, starting materials, and technologies used to generate and control reactive intermediates. In addition, the need for minimizing toxic waste and the respective disposal cost highlights how crucial it is to consider the recovery and reuse of the materials needed for a synthetic process. It is also very important to promote the use of biomass-derived chemicals that feature an intrinsically lower CO_2_ consumption.

Additionally, the pivotal role of catalysis is indisputable. Significant efforts are being directed towards the development of effective catalytic methodologies with safer and cheaper substrates where reactivity is achieved through catalysis that can replace the classically used, highly reactive species. While for immediate economical reasons the use of well-established methodologies based on homogeneous catalysis may be initially preferred, many efforts are directed toward the development of heterogeneous catalytic approaches, aiming at an easier recovery and better reuse of the catalyst.

Another key aspect of green chemistry, closely related to the chemical efficiency and efficiency of a protocol, is the technology behind the process. In fact, energy and time optimization are important factors. Increasing interest is being directed towards the development of innovative mixing and heating technologies that, individually or in combination, may furnish an innovative solution for controlling the safety and the reactivity of a chemical process and may facilitate the recovery and reuse of the materials used, which contribute to minimizing the energy consumption and increasing the overall efficiency of a process. Flow chemistry, microwave or ultrasonic irradiation, and mechano-chemistry are just a few representative examples of research platforms being independently developed, but all offer innovative tools for realizing chemically and environmentally efficient processes. Representative examples of these directions have been the subject of other excellent Thematic Series in the *Beilstein Journal of Organic Chemistry*, including “Strategies in asymmetric catalysis” by Tehshik P. Yoon [6], “Organometallic chemistry” by Bernd F. Straub and Lutz H. Gade [7], “C–H functionalization/activation in organic synthesis” by Richmond Sarpong [8], “Bifunctional catalysis” by Darren J. Dixon [9], “Sustainable catalysis” by Nicholas J. Turner [10], and “Organic synthesis using photoredox catalysis” by Axel G. Griesbeck [11], proving that green chemistry and sustainability can be approached from many different perspectives.

The breadth of chemical and technological innovations makes the definition of novel metrics for the evaluation of the quality of a new process in the field of green chemistry necessary. A key aspect of green chemistry is in fact the comparison of the different strategies available by considering as many experimental aspects as possible. Of course the most important feature to be evaluated is the correct measure of the waste generated, which is derived from both the synthetic strategy and the technology used. The fundamental role of green metrics is to evaluate the modern classification of chemical transformations in relation to the potential or actual pollution produced. In some cases, such as calculating the waste associated with the mass of the material used, this is easily evaluated. However, it may be more difficult to compare energy, time, labor costs, and other variables of a process. Certainly, innovation is the most important goal of green chemistry, but it is also the most difficult feature to measure and evaluate. Novel chemistry and innovative technologies are needed for the development of future, sustainable, chemical production. To reach this goal, both fundamental research, as well as the ability to translate the innovation into real world applications, should be combined.

Organic chemistry, with its kaleidoscope of interests and applications, offers the arena where countless opportunities exist to effectively contribute to the development of green chemistry. Journals dedicated to the field of organic chemistry, such as the *Beilstein Journal of Organic Chemistry*, represent an ideal medium for disseminating scientific efforts in this context. This Thematic Series, “Green chemistry”, collects original research and review articles, where an obviously limited but highly exemplificative portion of the broad field of green chemistry is described.

Luigi Vaccaro

Perugia, November 2016
